# Role of phenotypic plasticity and population differentiation in adaptation to novel environmental conditions

**DOI:** 10.1002/ece3.1607

**Published:** 2015-08-22

**Authors:** Sergei Volis, Danara Ormanbekova, Kanat Yermekbayev

**Affiliations:** 1 Key Laboratory for Plant Diversity and Biogeography of East Asia, Kunming Institute of Botany, Chinese Academy of SciencesKunming, 650204, China; 2 Department of Agricultural Sciences, University of BolognaVia Zamboni, 33, 40126 Bologna, Italy; 3 Institute of Plant Biology and Biotechnology45 Timiryazev St., Almaty, 050040, Kazakhstan

**Keywords:** Adaptation, climate change, emmer wheat, peripheral populations, phenotypic plasticity, phenotypic selection, species range

## Abstract

Species can adapt to new environmental conditions either through individual phenotypic plasticity, intraspecific genetic differentiation in adaptive traits, or both. Wild emmer wheat, *Triticum dicoccoides*, an annual grass with major distribution in Eastern Mediterranean region, is predicted to experience in the near future, as a result of global climate change, conditions more arid than in any part of the current species distribution. To understand the role of the above two means of adaptation, and the effect of population range position, we analyzed reaction norms, extent of plasticity, and phenotypic selection across two experimental environments of high and low water availability in two core and two peripheral populations of this species. We studied 12 quantitative traits, but focused primarily on the onset of reproduction and maternal investment, which are traits that are closely related to fitness and presumably involved in local adaptation in the studied species. We hypothesized that the population showing superior performance under novel environmental conditions will either be genetically differentiated in quantitative traits or exhibit higher phenotypic plasticity than the less successful populations. We found the core population K to be the most plastic in all three trait categories (phenology, reproductive traits, and fitness) and most successful among populations studied, in both experimental environments; at the same time, the core K population was clearly genetically differentiated from the two edge populations. Our results suggest that (1) two means of successful adaptation to new environmental conditions, phenotypic plasticity and adaptive genetic differentiation, are not mutually exclusive ways of achieving high adaptive ability; and (2) colonists from some core populations can be more successful in establishing beyond the current species range than colonists from the range extreme periphery with conditions seemingly closest to those in the new environment.

## Introduction

Plant responses to rapid environmental changes became one of the most important questions for evolutionary biology because of global change concerns. Species adaptive ability can be due to either individual phenotypic plasticity or intraspecific genetic differentiation to local environment in adaptive traits, or to both (Potvin and Tousignant 1996; Davis et al. 2005; Nicotra et al. 2010; Hendry et al. 2011; Reed et al. 2011).

When environmental factors imposing selective pressures are known, predicting changes to natural selection requires identifying traits (as well as their combinations) that will be targets of this selection, and determining role of phenotypic plasticity in trait selection. Then, the next step is establishing a relationship between environmental factors of interest (such as climate change), phenotypes, and fitness.

Because environment often determines both expression of genetic variation in phenotypes and the strength of selection, it is important to obtain estimates of selection intensity and trait values across a range of environmental variation of interest (Etterson 2008).

Both, fitness of different population phenotypes and expression of phenotypic differences between populations will be environment dependent if the phenotypes are under varying selection. Therefore, in estimating plant responses to predicted climate change, one needs to account for population differences in their range position and associated environmental differences. Response of populations at the range edge having extreme climatic conditions may differ from response of populations away from the edge having more benign conditions.

In this study, we analyzed role of plasticity and population genetic differentiation in adaptation to novel conditions. Choosing quantitative traits to study, we focused primarily on the onset of reproduction and maternal investment, which are traits that are closely related to fitness and presumably involved in local adaptation in the studied species (Volis et al. 2014a). Traits related to the timing of life-history transitions, such as timing of reproduction, are among those that are expected to experience the strongest selection as climate changes (Bradshaw and Holzapfel 2008). In annual plants, a switch from vegetative to reproductive stage in a proper time is one of the major determinants of plant reproductive output and is directly affected by the availability of limiting resources (Lacey 1986; Fox 1990; Arntz and Delph 2001; Sherrard and Maherali 2006). Plasticity in maternal investment is also of great importance because changes in fruit number and quality directly influence plant fitness. The total reproduction in plants comprises two components, the number of flowers developed into seeds and the weight of individual seeds. Thus, a measure of fecundity that estimates fitness must incorporate both reproductive components, and the total weight of mature seeds produced by a plant appears to be the best estimate of its fitness (Volis et al. 2004; Volis 2009). The fecundity can be optimized in several ways, either by maximizing the number of flowers/seeds in an inflorescence, the number of inflorescences, or seed size. Because the amount of resources available to the plant is limited, these components should trade off with each other, although the trade-offs may be environment specific.

Our scientific question was adaptation to conditions not currently experienced by the species but with high probability of encounter in the near future due to the global climate change. The effects of global climate change are predicted to be especially severe for Mediterranean-type ecosystems due to an intensification of their already limiting conditions for plant regeneration (Schröter et al. 2005). The predicted climate change in the Mediterranean basin includes both increasing aridity (i.e. higher temperatures, lower rainfall, and greater potential evapotranspiration), and greater frequency of extreme drought conditions (Gao and Giorgi 2008; Giorgi and Lionello 2008). Because a gradient of aridity determines distribution limits of many species in the Mediterranean basin, populations from a species periphery in this region often occupy the extremes of such gradient. Wild emmer wheat, *Triticum turgidum* L. ssp. *dicoccoides* (hereafter *T. dicoccoides*) is an annual grass with major distribution in Eastern Mediterranean region extending into southeastern Turkey, Iraq, and Iran. The species northern geographic limit is determined by low winter temperatures and southern limit by low precipitation (Willcox 2005; Özkan et al. 2011). Under predicted rapid aridification many grass-covered areas in the Mediterranean basin may transit to bare ground conditions (Zeng and Yoon 2009; Anav and Mariotti 2011), and the emmer wheat southern edge populations will experience conditions more arid than in any part of the current species distribution.

Genetic population differentiation (Fahima et al. 2002; Ozbek et al. 2007; Özkan et al. 2011) and population local adaptation (Volis et al. 2014a) have been reported in this species. Introduction beyond the current range of *T. dicoccoides*, in the Negev desert having around 200 mm of annual rainfall revealed inferiority of genotypes from the southern arid range population to those from the core population in spite of detected for these populations local adaptation (Volis et al. 2014a). To understand role of population origin in selective response to extreme aridity via either plasticity or population genetic differentiation, we used several populations of *T. dicoccoides* of different range position. We investigated how the mean trait value and pattern of trait correlations (phenotypic integration sensu Pigliucci and Kolodynska 2006) depend on population origin and growing conditions. We used path analysis to model causal interactions among phenological and morphological traits, and then analyzed how the paths were affected by growing conditions in which we manipulated water availability. We hypothesized that the population showing superior performance under novel environmental conditions will either be genetically differentiated in quantitative traits or exhibit higher phenotypic plasticity than the less successful populations. Surprisingly, the most successful population was both genetically differentiated from the other populations and had the highest plasticity.

## Methods

### Study species and sampling

**Figure 1:**
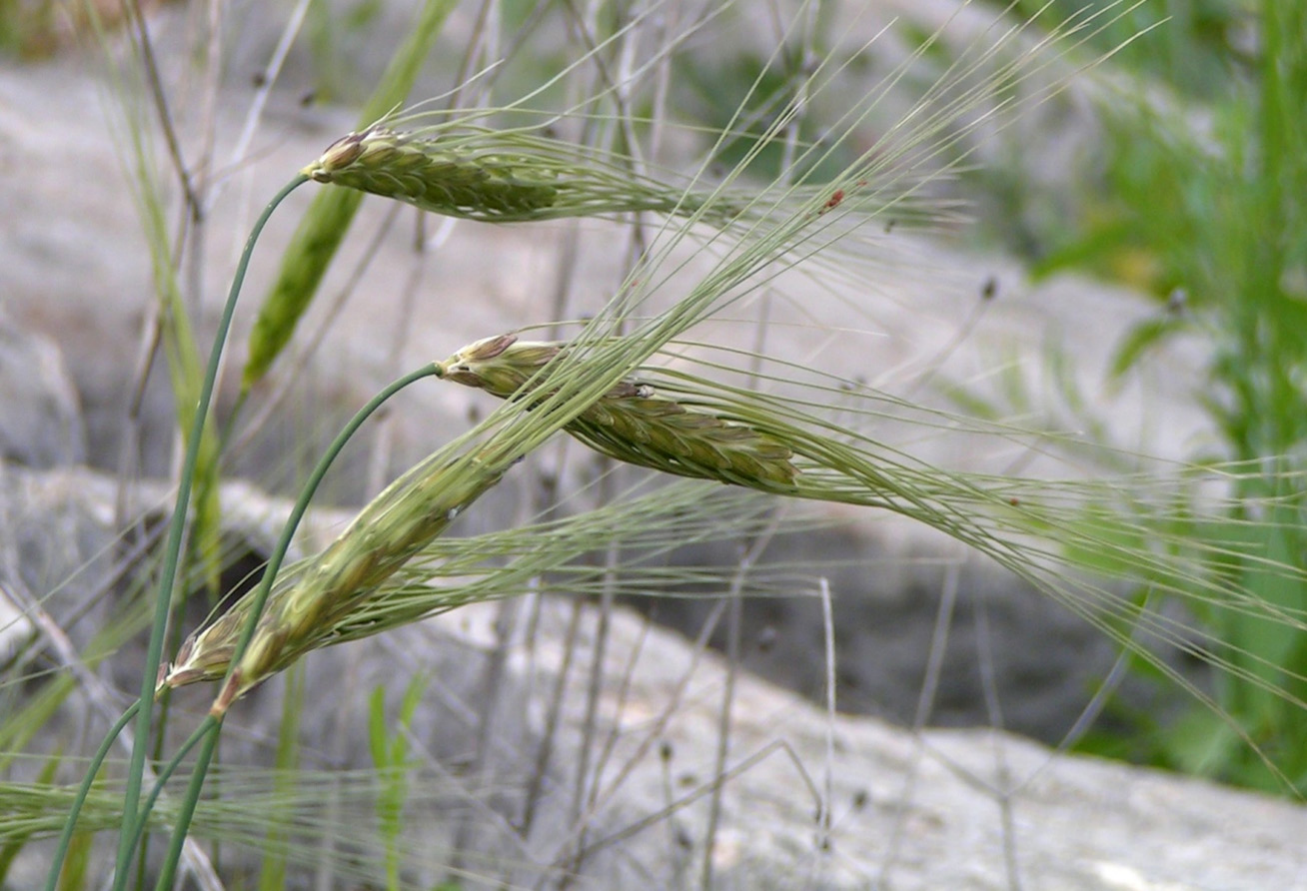
Wild emmer wheat (*Triticum turgidum* L. ssp. *dicoccoides*) in its natural environment.

Wild emmer wheat, *Triticum dicoccoides* is a predominantly self-pollinating annual grass (Fig.1) that is found in habitats with annual precipitation ranging 300–over 1300 mm at altitudes between −100 and 1400 m (Feldman and Sears 1981; Feldman and Kislev 2007). We have chosen two populations representing the species distributional core in the Upper Jordan Valley catchment area, and two populations from the two opposite edges of species distributional range. Ammiad conservation site from which two of the populations are derived (Ammiad Karst, latitude 32.923, longitude 35.526, and Ammiad North, latitude 32.918, longitude 35.532) is located north of the Sea of Galilee at altitudes between 240 and 350 m above sea level (Anikster and Noy-Meir 1991). It features a typical Mediterranean climate with an average annual rainfall of 580 mm (±151 mm SD). Ammiad North (N) is located on a moderate north-facing slope at an elevation of 260–280 m with relatively low rock cover (20–60%). Ammiad Karst (K) is on a steep south-facing slope of rockier micro-relief (40–80% rock cover) at 320–340 m above sea level. Mount Hermon (MH, latitude 33.287, longitude 35.764) population is the northern-most population that was found in Israel. The site is located on south-facing slope of the mountain at an elevation of 1450 m. The mean annual rainfall is more than 1300 mm (at Majdal Shams, few kms apart), and the climate is much cooler then in Ammiad (the area is covered with snow during winter months). Har Amasa (HA, latitude 31.352, longitude 35.111) population is the southern-most population that was found in Israel 19 Km south of Hebron and on the edge of Judean desert. It was found at an elevation of about 900 m and on east-facing slope. The climate is steppical and the mean annual rainfall is assumed to amount to <300 mm (383 mm was measured in Daharyyia, about 15 Km northwestwards on Judean Mountains). The plants grow in soil pockets between large rocks micro-relief.

We are fully aware of the drawbacks of a sampling design with no replication. However, there is only population of wild emmer wheat in a location with <400 mm rainfall (true species periphery) making replicated sampling design impossible. Sampling was conducted in 2007. Because one meter is a distance of major seed dispersal in emmer, in each of the four populations, the sampled plants were separated by more than 1 m to insure high probability of sampling genetically unrelated individuals. From each sampled plant, we took a separately bagged spike.

### Greenhouse experiment

The experiment aim was to study selective responses of accessions (= genotypes) from the four populations to simulated drought stress in an extremely arid environment vs. favorable water conditions. Because of high selfing (Volis et al. 2014b), progeny of each accession can be considered genetically identical. The experiment was conducted in winter 2010–2011. In this experiment, the plants were grown in a greenhouse at the Bergman Campus, Beer Sheva. Beer Sheva is located in the Northern Negev desert (annual rainfall 205 mm). Prior to the experiment, all the accessions were grown under uniform conditions in 2009–2010 in the same greenhouse as above to reduce the maternal effect. The seeds with reduced maternal effect were simultaneously germinated in an incubator at 24°C and transferred into 3-L pots arranged in a greenhouse. Within each water treatment, the plants were completely randomized. The pots were filled with the commercial potting mixture. The number of accessions for populations MH, K, N, and HA was 56, 56, 54, and 65, respectively. Each accession was represented by two plants that received one of two water treatments during the experiment, viz. the accessions were not replicated within the treatments. In the first treatment (high water), the plants received amount of water equivalent to 1058 mm of rainfall. This was not a predetermined amount and the latter resulted from the high evaporation rate in the greenhouse. Watering during the experiment was conducted regularly (twice a week) through drip-irrigation system to keep plants in a good condition, with no intent to imitate any particular environment encountered by the study species. In the second treatment (low water) the amount of water supplied to the plants was kept at minimum necessary for plants to survive and reproduce. Because of the high evaporation rate in the greenhouse due to different soil and ambient temperatures as compared with natural conditions, the amount of water supplied (equivalent to 460 mm of rainfall) is impossible to convert into naturally occurring precipitation but plants were showing signs of suffering from drought (low turgor and wilting of leaves) during the whole experiment. Water was applied from November to April to mimic the natural pattern of rainfall in the eastern Mediterranean region. The measured quantitative traits included flag and penultimate leaf length and width (FLL, PLL, FLW, and PLW), spike length (SPL), awn length (AWL), and number of spikelets in a spike (NSS), number of days to awning (DAW) and seed maturation (DMT). At senescence, mean spikelet weight (SWT) was obtained from the total number of spikelets (TSN) and total seed mass per plant (TSM). The traits were categorized into three categories: phenological (DAW and DMT), reproductive (FLL, PLL, FLW, PLW, SPL, AWL, NSS, and SWT), and fitness (TSN and TSM).

### Data analysis

A repeated-measures analysis of variance with one grouping factor (plant origin) and one within-group factor with unordered levels (treatments) was performed to assess univariate environmental, genetic, and interaction effects. An accession (= genotype) was not replicated. This analysis was the most appropriate to test the overall treatment effects (phenotypic plasticity), population effects (genetic variation for population trait means across treatments), and treatment * population interactions (genetic variation for plasticity).

A canonical discriminant analysis was used to estimate the overall plastic responses of plants of four origins to two water treatments. We used discriminant analysis because in this technique new variables are created to maximize the variation between groups relative to the variation within groups (Gurevitch 1992; Volis et al. 2002b; Volis 2009). As adaptation to a stress by plants of different origin may be achieved by differential trait and trait complex plasticity, the estimation of overall plasticity in this study was carried out separately for the three trait categories: phenology, reproductive, and fitness. This was performed by calculating the Mahalanobis distances between population centroids at two different levels of the same factor (water amount).

The trait selection effects in the experiment were calculated for each population separately. Relative fitness was estimated as the total weight of mature spikelets produced by a plant relative to the maximum observed under this treatment total weight of mature spikelets per plant. This measure of fitness is preferred over the total number of spikelets per plant as spikelet weight in annual grasses varies greatly among populations and individuals with a trade-off between number of spikelets and individual spikelet weight (Volis et al. 2004; Volis 2009). Selection differentials (*α*) were calculated as a covariance of relative fitness with the standardized traits. Directional selection gradients (*β*) were calculated as a partial linear regression coefficient of relative fitness on the standardized traits.

Path analysis was used to visualize the complex relationship between multiple traits and individual fitness under different environmental conditions (Kingsolver and Schemske 1991). In this analysis, the path diagram shows an explicit system of linear paths depicting the causal regulating mechanisms. We fitted a model with three hierarchical levels. In this model, phenology (days to awning) directly influence three reproductive traits (number of spikes, spikelets per spike, individual spikelet weight), and the latter, in turn, directly affect fitness estimated by total weight of spikelets. The model allowed for correlated error variables of the reproductive traits, as individual plants may tend to show above-average or below-average values for several of these fitness component traits simultaneously due to factors that are not explicitly specified in the diagram. In all population × environment combinations, model chi-squared values were nonsignificant, indicating good agreement of the proposed model with the actually observed covariances among traits. The path analysis was performed for each treatment/population separately using AMOS (Arbuckle 2006).

## Results

### Reaction norms and plasticity of traits

A repeated-measures analysis of variance showed high phenotypic plasticity in all the traits in response to the two water treatments (Table1). The population means across the water treatments were significantly different (i.e., there was significant genetic variation among populations) in all traits. A significant population * treatment interaction, which indicates interpopulation genetic variation for plasticity, was found in all the traits, except for flag leaf width and spike length.

**Table 1:** Repeated measures analysis of variance for the response to two water treatments in four populations of *T. dicoccoides*. The treatment effect estimates extent of phenotypic plasticity and treatment × population interactions indicate the inter-population genetic variation for plasticity

		Source of variation		
Trait	Type of trait	Population (df = 3)	Treatment (df = 1)	P × T (df = 3)
Days to awn appearance	Phenology	79.5***	18.0***	13.4***
Days to maturation	Phenology	39.6***	3.3 ns	14.3***
Flag leaf length	Reproductive	50.3***	299.8***	24.8***
Penultimate leaf length	Reproductive	90.6***	471.3***	21.9***
Flag leaf width	Reproductive	66.0***	60.7***	0.3 ns
Penultimate leaf width	Reproductive	151.3***	184.8***	3.9**
Awn length	Reproductive	33.7***	35.3***	3.0*
Spike length	Reproductive	27.4***	76.3***	1.3 ns
Spikelets per spike	Reproductive	50.9***	147.2***	3.2*
Spikelet weight (mg)	Reproductive	306.1***	59.7***	9.6***
Total seed weight (g)	Fitness	170.4***	702.1***	63.1***
Total seed number	Fitness	28.9***	347.9***	27.3***

^*^P <* *0.05; ^**^P <* *0.01; ^***^P <* *0.001, ns not significant. df error = 220.

**Figure 2:**
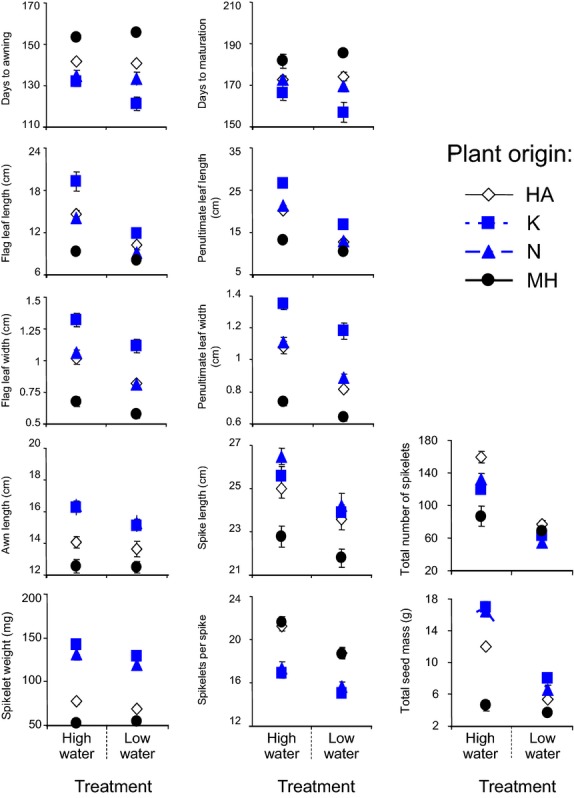
The population means (±SE) for 12 quantitative traits under two water treatments.

The population across-treatment reaction norms (Fig.2) show the following patterns of plasticity in the three trait categories:

1. Phenology: There was a difference in pattern of plasticity among populations. Water stress causes advance in the onset of reproduction in the K population, and delay in the MH population, with no effect on the N and HA populations. There was an advance in maturation in the two core populations, K and N, and delay in the two edge populations, MH and HA.
2. Reproductive traits: The differences in plasticity among populations were minor. Water stress either decreased size and number of reproductive traits (spikes, spikelets and associated flag and penultimate leaves) or had no effect on them.
3. Fitness: The reaction norms of the K, N and HA populations were highly similar and distinctly differed from that of the MH population.

**Figure 3:**
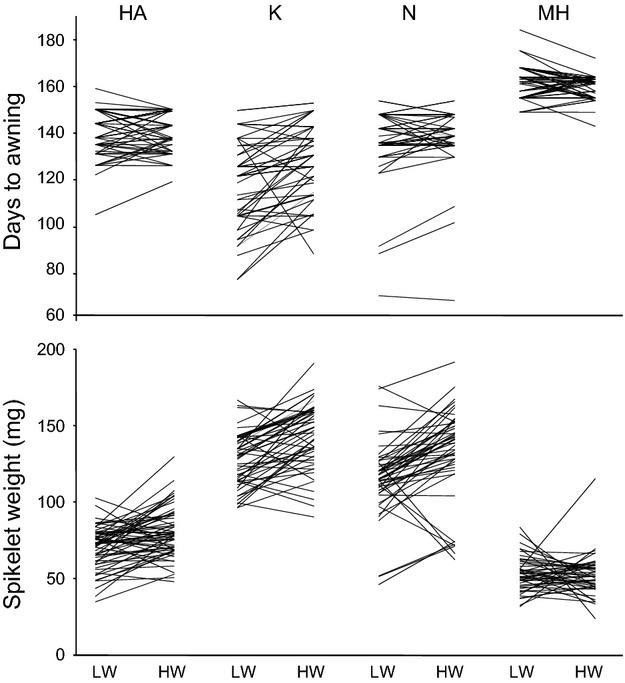
Individual reaction norms for days to awning and spikelet weight in four populations of emmer wheat. Each line depicts a unique genotype values under low water (LW) and high water (HW) treatment.

There was substantial intrapopulation variation in plasticity with crossing reaction norms (Fig.3).

The amount of plasticity in response to water stress measured by distance between centroids in the space created by the first two discriminating axes was higher in the K population than in the other three populations for phenological (1.2 vs. 0.2, 0.3 and 0.1), and reproductive traits (10.9 vs. 6.9, 8.5 and 4.5; Mahalanobis distances). Plasticity in fitness traits was decreasing from N to K, then to HA and MH plants (12.2, 10.7, 7.7, and 0.2, respectively).

### Selection analysis

#### Selection differentials

The selection differentials differed between the two water environments for intensity but not for direction of selection in most of the traits (with one exception) (Table2). Earlier-flowering and earlier-maturing K, N and MH plants had higher fitness under both water treatments, and HA plants had higher fitness under HW but not LW. Plants with larger flag and penultimate leaves, spikes, spikelets and spikelet awns had an advantage in at least one of the two water environments while plants with smaller reproductive traits had no advantage in any water environment.

**Table 2:** Standardized linear selection differentials for ten traits measured on plants of different population origin grown under two water treatments. Correlation coefficients significant after sequential Bonferroni adjustment are in bold

	Standardized selection differentials							
	HA		K		N		MH	
	HW	LW	HW	LW	HW	LW	HW	LW
Days to awn appearance	−0.37**	0.32*	−0.42**	−0.13	−0.34*	−0.36**	−0.31*	−0.54***
Days to maturation	−0.25	0.24	−0.38**	−0.11	−0.28*	−0.32*	−0.41**	−0.51***
Flag leaf length	0.11	0.30*	0.38**	0.07	−0.02	0.22	0.33*	0.44**
Flag leaf width	0.12	0.34**	0.21	0.16	0.15	0.33*	−0.05	0.51***
Penultimate leaf length	0.34**	0.26*	0.24	0.16	0.13	0.21	0.18	0.37**
Penultimate leaf width	0.08	0.27*	−0.14	0.03	0.23	0.32*	0.16	0.37**
Awn length	0.34**	0.38**	0.62***	0.08	0.39**	0.49***	0.36*	0.46***
Spike length	0.23	0.41**	0.55***	0.13	0.29*	0.56***	0.37*	0.63***
Spikelets per spike	−0.07	0.00	−0.33*	0.11	−0.25	0.05	−0.24	0.18
Spikelet weight	0.33**	0.54***	0.67***	0.27	0.46***	0.55***	0.11	0.38**

*P <* *0.05; **P <* *0.01; ***P <* *0.001.

#### Directional selection gradients

Multivariate selection analysis revealed direct positive effect of flowering time on fitness in HA population under LW, while in all other populations under both water treatments time to flowering and maturation had no effect (Table3). Spikelet weight had positive effect on plant performance in HA, N and MH plants under LW and in K under HW. Penultimate leaf length and spikelets per spike had positive effect, and awn length had negative effect on fitness under HW, and flag leaf length had negative effect on fitness under LW in HA population. In N population, flag leaf length had positive effect on fitness under LW.

**Table 3:** Standardized linear selection gradients (±SE) for ten traits measured on plants of different population origin grown under two water treatments. Correlation coefficients significant after sequential Bonferroni adjustment are in bold

	Standardized selection gradients							
	HA		K		N		MH	
Traits	HW	LW	HW	LW	HW	LW	HW	LW
Days to awn appearance	−0.28 ± 0.24	0.83 ± 0.26**	−0.39 ± 0.34	−0.77 ± 0.60	−0.79 ± 0.53	−0.35 ± 0.39	0.08 ± 0.24	−0.17 ± 0.19
Days to maturation	0.26 ± 0.21	−0.33 ± 0.24	0.11 ± 0.32	0.56 ± 0.53	0.66 ± 0.56	0.13 ± 0.39	−0.39 ± 0.24	−0.41 ± 0.18*
Flag leaf length	−0.58 ± 0.23*	−0.59 ± 0.19**	−0.28 ± 0.26	−0.57 ± 0.36	−0.42 ± 0.22	0.04 ± 0.18	0.46 ± 0.27	−0.49 ± 0.22*
Flag leaf width	0.41 ± 0.25	0.42 ± 0.17*	0.17 ± 0.24	0.36 ± 0.26	0.05 ± 0.18	0.11 ± 0.20	−0.06 ± 0.15	0.20 ± 0.16
Penultimate leaf length	0.72 ± 0.23**	0.22 ± 0.17	0.22 ± 0.26	0.08 ± 0.32	0.30 ± 0.20	−0.16 ± 0.17	−0.64 ± 0.31	0.44 ± 0.15**
Penultimate leaf width	−0.33 ± 0.22	−0.05 ± 0.12	−0.43 ± 0.15*	−0.16 ± 0.27	0.27 ± 0.21	−0.19 ± 0.20	0.02 ± 0.26	0.06 ± 0.11
Awn length	−0.69 ± 0.25**	0.31 ± 0.27	0.05 ± 0.21	−0.10 ± 0.31	0.27 ± 0.38	0.97 ± 0.33**	0.43 ± 0.43	0.60 ± 0.25*
Spike length	0.86 ± 0.30**	0.022 ± 0.31	0.21 ± 0.26	0.35 ± 0.36	−0.17 ± 0.45	−0.76 ± 0.34*	−0.26 ± 0.45	−0.44 ± 0.24
Spikelets per spike	0.57 ± 0.17**	0.20 ± 0.16	−0.02 ± 0.15	0.42 ± 0.21	−0.06 ± 0.24	0.12 ± 0.15	−0.43 ± 0.27	−0.04 ± 0.14
Spikelet weight	0.26 ± 0.15	0.50 ± 0.13***	0.42 ± 0.13**	0.44 ± 0.20*	0.37 ± 0.19	0.58 ± 0.18**	0.31 ± 0.18	0.51 ± 0.14**

*P <* *0.05; **P <* *0.01; ***P <* *0.001.

#### Path analysis of selection

**Figure 4:**
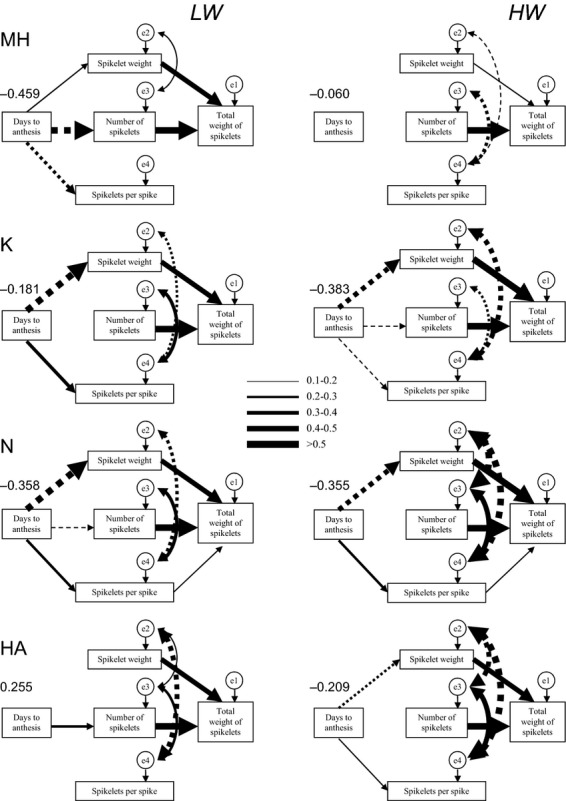
Path analysis of selection in the four populations under low water (LW) and high water (HW) treatments. Line thickness indicates magnitude of path coefficients. Solid and dashed lines denote positive and negative relationship, respectively. The value above the box with days to anthesis denotes the total effect of this trait on total weight of spikelets.

Visual inspection of path coefficients (Fig.4) revealed how onset of flowering affected fitness via intermediate reproductive traits, spikelets per spike, spikelet weight, and number of spikelets, and how the latter traits affected fitness directly and indirectly.

The individual spikelet weight and number of spikelets per plant had strong direct positive effect on fitness in both water environments while direct effect of spikelets per spike on fitness was either weak or absent. Other path coefficients were population specific and treatment specific (Fig.4).

Early flowering was advantageous in all populations under high water treatment and in the three populations under LW, as indicated by the total effect of this trait on fitness (Fig.4). However, in HA population, the direct effect of onset of flowering on fitness under LW was positive, that is, delayed flowering was selected for. Both, direct, and indirect effects of flowering time on fitness in the two core populations, K, and N, were highly similar and were little affected by amount of water supplied to the plants. Although in these populations early flowering plants were producing less spikelets per spike, they had larger spikelets, and, as a result, had higher total seed mass. In MH population, flowering time had very weak negative effect on fitness under HW but very strong negative effect under LW. The latter effect was due to a negative effect of flowering time on number of spikelets produced despite a positive effect on individual spikelet weight. In HA population, flowering time under HW had a positive effect on individual spikelet weight but negative effect on number of spikelets in a spike with the total effect being negative. Under LW, the total effect of flowering time became positive because of its positive relationship with number of spikelets produced and disappearance of the above two effects.

Two reproductive traits, individual spikelet weight and number of spikelets in a spike were negatively correlated in all four populations under HW and in the three populations under LW. In MH population, these two traits were not correlated under LW. The intercorrelations between the three reproductive traits were similar in the K, N, and HA plants, with little effect of water treatment. In contrast, in MH plants the correlations among these traits were distinctly different under HW and LW, with no relationship between individual spikelet weight and number of spikelets in a spike under water stress.

## Discussion

### Reaction norms and plasticity of traits

The two experimental water treatments induced a significant plastic response in most of the studied phenotypic traits. This was expected, as low precipitation is one of the major factors limiting *T. dicoccoides* distribution (Willcox 2005; Özkan et al. 2011). All the traits exhibited genetic variation among populations and most of the traits showed a different interpopulation pattern of plasticity across treatments. As the MH and HA plants were previously found to be adapted to their local environment, and the K and N plants were found to be adapted to the conditions of their common environment (Volis et al. 2014a), these two sources of genetic variation among populations MH, HA and combined K and N should be ascribed to predominant effect of selection, while any differences between K and N appear to be due to nonselective processes.

The K plants had the highest overall plasticity either alone (phenological and reproductive traits) or together with N plants (fitness traits) in a response to water stress. These results only partly agree with the hypothesis that plants originating in environments with greater variation and unpredictability are more plastic (Schlichting 1986; Debat and David 2001; Alpert and Simms 2002). In Israel, water is the main limiting and fluctuating resource in this area that creates a severe north-south aridity gradient, with a negative exponential relationship between annual rainfall amount and its coefficient of variation (*R*^2^ = 0.79, *n* = 25; see Fig.1 of Volis et al. 2002a). Although the MH ecotype inhabiting the most stable and predictable with respect to amount and timing of rainfall both within and among seasons mountain environment was the least plastic, the desert ecotype inhabiting the most rainfall unpredictable semi-arid environment showed lower plasticity in all trait categories in response to water stress than the K and N plants from more predictable Mediterranean environment. In a methodologically similar study, Dryer with colleagues (Dyer et al. 2012) found higher phenological plasticity in a mesic (forest) than a xeric (desert) population of an annual grass *Bromus tectorum*. Their explanation was that, although total rainfall is predictably greater at the mesic site, the early onset of dry conditions is much more predictable at the xeric site. This can explain why in our study plants from semi-desert site (HA) exhibited lower plasticity in start or flowering than plants from mesic (K and N) sites. Similarly, a difference in plasticity in other (reproductive and fitness) traits between the two sites can reflect a difference between fixed vs. opportunistic response to predictability of dry conditions (Dyer et al. 2012).

Irrespective of growth conditions, the K and N plants from mesic Mediterranean environment had distinctly larger spikes and spikelets but with smaller number of spikelets per spike than plants of mountain and semi-arid origin. Although the total number of seeds produced by the plants of Mediterranean origin did not differ from or was smaller than one produced by the plants of other origin, the Mediterranean-origin plants were superior under all growing conditions in total reproductive biomass. This suit of traits possessed by the plants from the studied Mediterranean location (Ammiad) that distinguishes them from plants from other climatic zones of Israel is remarkably similar to what was found in this location for two other annual grasses, *Hordeum spontaneum* and *Avena sterilis* (Volis et al. 2002b; Volis 2007). The plants of mountain origin (MH) differed from the other origins in having the smallest spikes and spikelets, short and narrow leaves, low reproductive biomass and very late reproduction. Again, these features are common to what was found for *H. spontaneum* and *A. sterilis* plants from the same location (Volis et al. 2002b; Volis 2007). Low reproductive biomass and late reproduction were also found in *T. dicoccoides* originating from high altitude locations having low temperatures by Peleg et al. 2005). The semi-arid plants (HA) were similar to Mediterranean plants (mostly to plants of N origin) in leaf traits, start of reproduction and total reproductive biomass, but, like mountain plants, had small spikes with small but numerous spikelets. Surprisingly, the HA plants from semi-arid environment flowered later under both water treatments than plants from the two mesic sites. Although the semi-arid plants were previously found to be locally adapted (Volis et al. 2014a), it can be that a specific combination of traits in this population reflects both, effect of local selection in traits under strong selection and some gene flow (at least in the past) from the species mesic core in traits experiencing weak local selection. The estimation of gene flow from the core Ammiad to the two peripheral populations using microsatellites revealed the 4*N*_*e*_*m* to be around unity from Ammiad to both MH and HA (S. Volis, D. Ormanbekova, K. Yermekbayev, M. Song, I. Shulgina, unpubl. ms.), which means a moderate historical gene flow from the core toward both the northern and southern periphery.

Strong interpopulation phenotypic differentiation in emmer wheat was accompanied by strong genetic (microsatellite) differentiation among the three populations (*F*_ST_ = 0.27), and the HA population was most differentiated genetically from the other populations (Volis et al., submitted).

### Selection for drought avoidance

*Triticum dicoccoides* is an annual grass inhabiting open Mediterranean vegetation, viz. it grows in a habitat with low precipitation throughout its short growing season (October – March), and rapid development and flowering before onset of summer drought is essential for survival and reproduction in these conditions. Selection for earlier flowering was detected in winter annuals from Mediterranin climate regions in a number of studies (Stanton et al. 2000; Rajakaruna et al. 2003; Peleg et al. 2005; Sherrard and Maherali 2006; Volis 2009). Interestingly, in all winter annual grasses studied (*T. dicoccoides* – this study and Peleg et al. 2005; *Hordeum spontaneum* and *Avena sterilis* – Volis 2009; *Avena barbata* - Sherrard and Maherali 2006) early flowering was favored in both, dry and well-watered environments. This may indicate a general positive physiological relationship of early start of reproduction with successful plant maturation in Mediterranean annual grasses.

For both core populations with mesic Mediterranean climate, under both water treatments onset of flowering had negative effect on plant fitness through its direct negative effect on spikelet weight. This regulating mechanism was also present in the population from arid edge under high but not low water supply. However, in the cold edge population under both water treatments and in the arid edge population under low water, the mechanism by which onset of flowering regulated fitness was different. These results illustrate how originally evolved in typical for the species core Mediterranean environment physiological regulating mechanism underwent changes under more stressful for the species environmental conditions. As both, cold edge and arid edge populations were previously found to be locally adapted (Volis et al. 2014a), these new regulating mechanisms probably constitute a part of the ecotypes' adaptive syndromes.

### Regulation of maternal investment

Regulation of maternal investment in *T. dicoccoides*, like other annual grasses (e.g., *H. spontaneum*, Volis et al. 2004) is achieved through adjustment of such reproductive traits as number of spikelets in a spike (at early stage) and weight of spikelets (at later stage of maturation). There was a general trade-off between these two traits indicated by their strong negative correlation under both water treatments. This trade-off can be explained by intraplant competition for resources, when increase in the number of spikelets per spike is compensated by decrease in their weight, with no change in the total weight of fertile spikelets. However, this trade-off was even more pronounced under high than low water supply, indicting a genetically determined rather than plastic trait relationship.

Despite a crucial role of functional relationship between number of spikelets in a spike and weight of spikelets in regulation of maternal investment in *T. dicoccoides*, there was a high variation in strength and even direction of other trait correlations among populations and growing conditions. This indicates high plasticity and environmental sensitivity of the character architecture in general and of the regulating mechanism of maternal investment in particular, in *T. dicoccoides*.

### Population adaptive ability and conservation implications

Effects of rapid climate change on vegetation communities and particular species have been studied predominantly by climate modeling and not experimentally (but see Etterson and Shaw 2001; Grime et al. 2000, 2008; Baez et al. 2013; Tielbörger et al. 2014). Our experimental study focused on effect of aridification on populations having different species range position. Previously, we reported the results of the introduction beyond the current range of *T. dicoccoides*, in the Negev desert having around 200 mm of annual rainfall (Volis et al. 2014a). In this introduction experiment, genotypes from the southern arid range population (HA) were inferior to genotypes from the core population (K) having more mesic Mediterranean climate, in spite of detected for the arid edge population local adaptation (Volis et al. 2014a). Here, we show that the core population K is the most plastic in all three trait categories (phenology, reproductive traits and fitness) and most successful among populations studied, in both experimental environments, including water stress conditions. At the same time this population showed local adaptation (viz. superior performance in its own but not other natural environments) in a reciprocal introduction test (Volis et al. 2014a). This means that the K ecotype is not a jack of all trades, if we consider the current species native environmental range. Thus, the species successful adaptation to new environmental conditions can be achieved without sacrificing phenotypic plasticity in favor of adaptive genetic differentiation or vice versa.

## Conclusions

Our results suggest that the two means of successful adaptation to new environmental conditions, phenotypic plasticity and adaptive genetic differentiation, are not mutually exclusive ways of achieving high adaptive ability. We also show that colonists from some (but not all) core populations can be more successful in establishing beyond the current species range than colonists from the range extreme periphery with conditions seemingly closest to those in the new environment. This should not be interpreted that peripheral populations in general will be less successful in adapting to changing environmental conditions than core populations, but implies caution in usage of peripheral populations in conservation projects.
